# In vitro hyperthermic effect of magnetic fluid on cervical and breast cancer cells

**DOI:** 10.1038/s41598-020-71552-3

**Published:** 2020-09-17

**Authors:** Anand Bhardwaj, Kinnari Parekh, Neeraj Jain

**Affiliations:** 1 Dr. K C Patel R&D Centre, Charotar University of Science and Technology (CHARUSAT), Changa, 388 421 India; 2 P D Patel Institute of Applied Sciences, Charotar University of Science and Technology (CHARUSAT), Changa, 388 421 India

**Keywords:** Molecular biophysics, Cancer therapy, Nanoparticles, Nanotechnology in cancer, Characterization and analytical techniques

## Abstract

Self-regulating temperature-controlled nanoparticles such as Mn–Zn ferrite nanoparticles based magnetic fluid can be a better choice for magnetic fluid hyperthermia because of its controlled regulation of hyperthermia temperature window of 43–45 °C. To test this hypothesis magnetic fluid with said properties was synthesized, and its effect on cervical and breast cancer cell death was studied. We found that the hyperthermia window of 43–45 °C was maintained for one hour at the smallest possible concentration of 0.35 mg/mL without altering the magnetic field applicator parameters. Their hyperthermic effect on HeLa and MCF7 was investigated at the magnetic field of 15.3 kA/m and frequency 330 kHz, which is close to the upper safety limit of 5 * 10^9^ A/m s. We have tested the cytotoxicity of synthesized Mn–Zn ferrite fluid using MTT assay and the results were validated by trypan blue dye exclusion assay that provides the naked eye microscopic view of actual cell death. Since cancer cells tend to resist treatment and show re-growth, we also looked into the effect of multiple sessions hyperthermia using a 24 h window till 72 h using trypan blue assay. The multiple sessions of hyperthermia showed promising results, and it indicated that a minimum of 3 sessions, each of one-hour duration, is required for the complete killing of cancer cells. Moreover, to simulate an in vivo cellular environment, a phantom consisting of magnetic nanoparticles dispersed in 1 and 5% agarose gel was constituted and studied. These results will help to decide the magnetic fluid based hyperthermic therapeutic strategies using temperature-sensitive magnetic fluid.

## Introduction

Magnetic fluid hyperthermia (MFH) is emerging as an effective, robust and feasible futuristic less-invasive cancer therapeutic option due to its minimal side effects compared to radio and chemotherapies^1–4^. The MFH based therapy includes administration of biocompatible magnetic fluid (MF) directly into the tumor which is placed under an alternating magnetic field with a frequency between 100 and 500 kHz. The magnetic nanoparticles (MNPs) sense the magnetic field and generate localized heating under the AC magnetic field via mechanisms of hysteresis and relaxation losses^5–7^. The aim of the MFH therapy is to achieve the hyperthermia temperature between 43 and 45 °C in minimum possible time and maintain it for a sufficient duration for the successful killing of cancer cells without affecting the surrounded healthy tissues. The dispersed magnetic particles in the tumor acts as a point source of heat, the power of which is directly proportional to the product of frequency and the square of magnitude of the applied magnetic field^8^. The heating ability of nanoparticles depend on the specific absorption rate (SAR) (W/g) which is a function of material composition and its micro-structural properties such as material type, particle size, size distribution, shape, dipolar interaction, surface functionalization, etc^9–11^.

Iron oxide nanoparticles specially magnetite and maghemite are mostly used for biomedical applications due to low toxicity index and their effects on well-known metabolic pathways^12^. Unfortunately, high SAR within a short span of time often causes overheating of the cells beyond 43 to 45 °C that leads to tissue necrosis, causing inflammation, which requires surgical intervention to remove inflamed tissue^13^. In addition, maintaining the hyperthermia window temperature using magnetite needs manual intervention of the magnetic field applicator. This can be avoided by developing self-controlled heating of the Mn–Zn ferrite MNPs by limiting their Curie temperature. Thus the heat dissipation from magnetic particles to its surrounding will automatically stop when the temperature of magnetic particles reach at its Curie temperature. In this way, the magnetic particles will act as a temperature control switch. The maximum dissipation temperature of these particles can be tuned by adjusting the Curie temperature of the particle through its chemical composition. Cancerous cells are more prone to be destroyed at a slightly lower temperature as compared to that of healthy cells. So the temperature-sensitive magnetic particles can selectively destroy the cancerous cells without affecting the healthy one. Furthermore, limiting the Curie temperature is expected to cause cell death through the mechanisms of apoptosis thereby providing a safeguard against tissue necrosis. By doping the composition with Zn^2+^, the Curie temperature can be reduced near to room temperature and with proper concentration of Zn^2+^ in MnFe_2_O_4_, the high SAR can be obtained.

In the case of auto-tunable Mn–Zn ferrites, reported are mostly available on material hyperthermia, with no study reported on the biological systems^14–17^. However, these studies indicate the potential suitability of this material for MFH with excellent magnetic responsibility, good heating and thermostatic ability, and biocompatibility. A single report by Attar and Haghpanahi^13^ demonstrated the effect of self-regulating temperature-controlled superparamagnetic iron oxide nanoparticles on colorectal, prostate and breast cancer cell lines leading to cell death. Looking to this, there is a wide opportunity to work with this material for MFH. Moreover, the effects of MFH at different time intervals, i.e., multiple sessions hyperthermia has solely been reported by Makridis et al.^18^, using Mn-ferrite nanoparticles only on osteosarcoma cell line and no study till date is available of the same effect using Mn–Zn ferrite.

The present study was conceived considering the potential of Mn–Zn ferrite nanoparticles over the presently available self-regulating temperature-controlled nanoparticles and lack of detailed investigations on biological systems. We report here for the first time, the synthesis and characterization of the auto-tunable Mn–Zn ferrite fluid and their hyperthermic effect on cervical and breast cancer cell lines HeLa and MCF7 respectively, at magnetic field of 15.3 kA/m and frequency 330 kHz which is near to upper safety limit of 5 * 10^9 ^A/m s^19^. The novelty of the work is to maintain the hyperthermia temperature of 43–45 °C for a longer duration by fixing the concentration of MNPs and the magnetic field within the safety limit. Additionally, the protocols of performing in vitro single and multiple sessions hyperthermia were also established. We have tested the cytotoxicity of synthesized Mn–Zn ferrite fluid using MTT assay which was validated by trypan blue dye exclusion assay, since this assay provides a naked eye view of the cell death. Moreover, as cancer cells are known to exhibit antineoplastic drug resistance, we also probed into the effect of multiple sessions hyperthermia on cell death using a 24 h window till 72 h by trypan blue assay. Further, to simulate an in vivo cellular environment a phantom consisting of MNPs dispersed in 1 and 5% agarose gel was constituted and studied.

## Materials and methods

### Synthesis of magnetic fluid

Mn_0.9_Zn_0.1_Fe_2_O_4_ nanoparticles were synthesized using 1 M solution of Mn^2+^ (9 mL), Zn^2+^ (1 mL) and Fe^3+^ (20 mL) as salt solutions and NaOH (80 mL) as base solution. The ratio of Mn to Zn was 9:1 while Mn to Fe was 0.9:2.0. The salts of appropriate molar ratio were mixed in base solution at 80 °C temperature and the mixture was stirred for 30 min at the same temperature. The impurities were removed using distilled water wash by magnetic sedimentation. Lauric acid was added to the particles as a surfactant which was then heated to 90 °C for 5 min to achieve stable magnetic fluid^20^. Density of synthesized magnetic fluid was measured using 10 mL gravity bottle. The parent fluid had 80 mg/mL concentration of magnetic particles which was diluted using distilled water to vary the concentration of the particles.

### Characterization of magnetic fluid

X-ray diffractometer, D2 phaser from Bruker containing Copper K_α_ source, with heating filament operating at 30 kV and 10 mA, generating characteristic X-rays of wavelength 0.154056 nm was used. Dried particles were grinded and passed through 400 mesh and then placed on the sample holder. Experiment was conducted in the angular range 2θ varied from 25° to 70° in step size of 0.05°. TEM images were obtained from JEOL, JEM 2100 operated at a 200 kV. The sample was prepared by diluting the fluid in aqueous medium, ultra-sonicated and a drop of fluid was placed on a carbon coated copper grid and dried overnight under vacuum. Thermo-gravimetric analyzer (TGA) model TGA-DSC-1 from METTLER, USA was used to study the percentage binding of surfactant on particle surface. The temperature was varied from 25 to 500 °C in steps of 10 °C per minute under an inert environment. Fourier transform infrared spectrometer (FTIR) model Nicolet from Thermo Scientific, USA was used to reveal the presence of surfactant and the nature of its binding with the particle surface. The pellet was made from the dried coated particles mixed with KBr. The wavenumber was varied from 400 × 10^2^ to 4000 × 10^2^ m^−1^. Magnetic properties of the fluid sample were measured at 300 °K using vibrating sample magnetometer (VSM) model 7404 from LakeShore, USA. The magnetic field was varied from 0 to 1 T. Particle Size Analyzer model S90 from Malvern Instruments Ltd., UK was used for DLS measurement which measures hydrodynamic size varying between 0.6 and 6000 nm. Respective dilutions were made in Milli-Q water with refractive index of dispersant as 1.33 and MNPs as 1.52. The measurement was carried out at 300 °K.

Induction heating model Easy Heat 8310 from Ambrell, USA was used to study the effect of magnetic hyperthermia on the cells. The setup consisted of induction heating copper coils, coil hood, power supply, a chiller to maintain temperature of coil and a biosafety cabinet for cell culture experiments to keep the cell lines isolated from the outside environment. The instrument provides frequency and current in the range of 330–340 kHz and 10–459 A, respectively. For induction heating experiments, the magnetic fluid was filled in a sample holder which was thermally insulated using rubberized cork sheet placed inside the heating coils. Alternating magnetic field was supplied by Ambrell power supply. The sample holder and induction coils were placed in a biosafety cabinet for cell culture experiments. Water was constantly circulated and the temperature of the coil was maintained through a water circulating chiller throughout the experiments. The water flow in the unit as well as copper pipe coil was maintained at 5.7 L/min in order to match the temperature of the coil with ambient temperature. The same was confirmed by measuring the temperature rise of water as a function of time at 459 A current. For the induction heating study of the magnetic fluid, a fixed magnetic field corresponding to the applied current in control panel was set and the sample holder containing magnetic fluid was placed between the induction coils. The temperature rise of the sample was measured using the fiber optic sensor dipped in the magnetic fluid that was placed in between the center of the coils. The initial temperature of 29–30 °C was fixed for all the samples before recording the temperature data as a function of induction heating time. The medium sized 2 × 2 turns helmholtz coil with inner diameter of 60 mm was used for the cell culture experiments where the magnetic field varied from 1.7 to 15.3 kA/m. The magnetic flux density varies along the z axis of the solenoid according to the formula^21^.

1 $$ H = \frac{NI}{{2L}}\left[ {\frac{{ - z + \frac{L}{2}}}{{\sqrt {\left( { - z + \frac{L}{2}} \right)^{2} + R^{2} } }} + \frac{{z + \frac{L}{2}}}{{\sqrt {\left( {z + \frac{L}{2}} \right)^{2} + R^{2} } }}} \right] $$

where N is the number of turns, I is the electric current intensity, L is the length of the solenoid, R is the solenoid radius and z is the distance from the center of the solenoid to the point on the axis where the magnetic field strength is calculated. The magnetic field strength values calculated using Eq. (1) is considered in the present work. Similar way of measuring and calculating magnetic field for Ambrell make 8 turn copper coil is done by Iacovita et al.^22^.

### Cell culture

The in vitro studies were carried out on cervical and breast cancer cell line HeLa and MCF7 respectively, obtained from National Centre for Cell Science, Pune, India. The cells were grown on 25 cm^2^ vented culture flasks (Corning, USA) using Eagle's Minimal Essential Medium (EMEM) (Thermofisher, USA) supplemented with 10% heat-inactivated fetal bovine serum (Thermofisher, USA), and 100 U/mL Penicillin, 100 µg/mL Streptomycin and 0.25 µg/mL Amphotericin B (Thermofisher, USA), in a 37 °C incubator with 5% CO_2_ and 95% relative humidity.

### MTT assay

Cytotoxicity of MNPs was assessed by MTT [3-(4, 5-dimethylthiazol-2-yl)-2, 5-diphenyltetrazolium bromide] assay. Briefly, 10^4^ HeLa/ MCF7 cells per well were seeded in triplicates in a 96-wells tissue culture plate and grown for 24 h followed by treatment with magnetic fluid diluted in EMEM in the concentration range varying from 0.75 to 0.03 mg/mL. Two sets of triplicates served as untreated controls.

After incubation the magnetic fluid was removed and the cells were washed thrice with phosphate buffered saline (PBS) to ensure complete removal of MNPs from the wells. Thereafter, 300 μl of media and 25 μl of MTT solution (5 mg/mL in PBS) was added to each well followed by 3 h of incubation. Subsequently, media containing MTT was removed and the formed crystal formazan were dissolved in 100 µl dimethyl sulfoxide. The MTT assay absorbance was measured at 570 nm on an ELISA plate reader (Molecular Devices, USA). After obtaining the IC_50_ value we utilized the same assay to report our preliminary results of 24 h hyperthermic effect on breast cancer cell line MCF7.

### Trypan blue (TPB) assay

Cytotoxic effect of MNPs was additionally determined using TPB assay after culturing HeLa cells on 35 mm culture dishes (Corning, USA). This assay was also used to determine cell viability subsequent to all hyperthermic effects on HeLa cells. Briefly, the cells were seeded at a density of 2.5 * 10^5^ cells/culture dish and treated for the time and doses as mentioned in MTT assay above. However, at the end of treatment, instead of adding MTT, the cells, after PBS wash and trypsinization by 0.25% Trypsin-EDTA (Thermofisher, USA), were stained with trypan blue solution and placed on hemocytometer for viability count under the microscope.

### Hyperthermia (HT) treatment

To examine the effect of magnetic hyperthermia by induction heating, HeLa and MCF7 cells were grown on 35 mm culture dishes, treated with 0.35 mg/mL magnetic fluid concentration for 24 h, and subsequently placed within 2 × 2 turns Helmholtz coils with 60 mm inner diameter and 330 kHz frequency (Ambrell, UK) in a wooden Class II/A2 biosafety cabinet. To avoid contamination, temperature measurement was performed within the Biosafety Cabinet which is widely used worldwide to avoid contamination during cell culture experiments. The biosafety cabinet is located in the Institute’s biosafety level II laboratory dedicated for cell culture and hyperthermia work where a constant temperature and pressure is maintained.Briefly, the method involved seeding 0.25 million cells in six culture dishes numbered as (1) control without MF and without HT; (2) cells without MF and 30 min HT; (3) cells without MF and 60 min HT; (4) cells with MF; without HT (5) cells with MF and 30 min HT; and (6) cells with MF and 60 min HT. Once the cells were approximately 80% confluence, MF was added to culture dishes 4, 5 and 6 only. The cells in all the dishes were further allowed to grow for the next 24 h. Subsequently, all the cells underwent induction heating except the cells of dishes 1 and 4 with respective durations. The magnetic field of 15.3 kA/m was set to achieve the temperature window of 43–45 °C, which was maintained throughout the treatment period. Subsequently, the cells were assessed for viability. The fiber optic sensor used for temperature measurement was wiped with 70% ethanol prior to dipping in the sample every time.

### Multiple sessions hyperthermia

The effect of multiple sessions hyperthermia was studied on HeLa cells. The concentration of magnetic fluid and HT duration was fixed at 0.35 mg/mL and one hour respectively. The experimental design consisted of 3 sets of culture dishes with four culture dishes in set 1, and 3 culture dishes in set 2 and 3. The culture dishes were numbered as (1) control cells without MF and without HT, (2) cells without MF but with HT, (3) cells with MF without HT and (4) cells with MF and HT. The culture dish 1 of set 1 served as control across all the sets.

**Figure 1:**
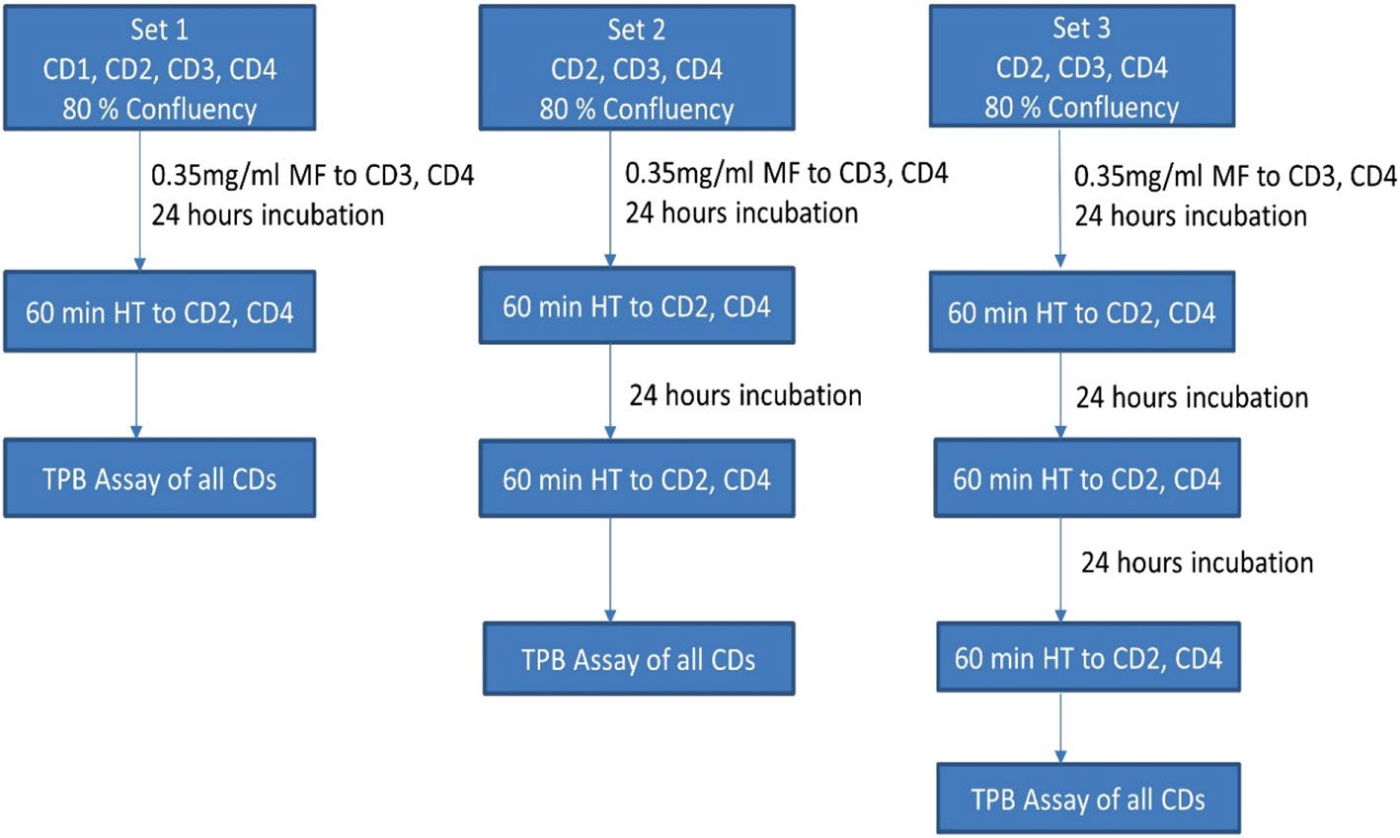
Flowchart of multiple sessions hyperthermia experiment. CD = Culture Dish, HT = Hyperthermia, MF = Magnetic Fluid, TPB = Trypan Blue.

Briefly, when the cells reached approximate 80% confluency, after seeding 0.25 million HeLa cells, MF was added to dishes 3 and 4, and incubated for 24 h along with rest of the dishes. After 24 h, cells in dishes 2 and 4 of all the sets underwent first HT session of 60 min duration. Subsequent to the HT session, all the cells of the first set of culture dishes were PBS washed and cell viability was assessed using TPB assay. The cells of second and third set were continued to grow for next 24 h. Afterwards, the cells of dishes 2 and 4 of second and third set underwent second HT session. Thereafter, the cells of second set were assayed for cell viability as per the procedure followed for first set of culture dishes. For the third HT session, cells of culture dishes 2 and 4 of the third set were continued to grow for next 24 h. Later, the cell viability was assessed of the third set. Overall, the cells of culture dishes 2 and 4 of first, second and third set received single, double and treble times hyperthermia treatment of one hour respectively. The cell viability was determined using TPB assay after comparing treated cells against the control cells that were neither exposed to MF nor to HT treatment. Figure 1 represents a flowchart of steps involved in multiple sessions hyperthermia. The results of each set were normalized against the untreated cells of culture dish 1 of the set 1.

### Statistical analysis

All the experiments were performed in triplicates. After obtaining mean and standard deviations, the data are expressed as mean values ± SE. The levels of significance were calculated using Student’s t-test and considered significant if the *p*-value was less than 0.05.

### Phantom agar system

The impact of HT on the tissue-mimicking phantom system was also studied using an agar-based gel system. Briefly, 1 and 5% agarose gel was prepared by boiling agarose powder in distilled water, and once the temperature of the solution reached 55 to 60 °C, the magnetic fluid of 0.35 and 0.75 mg/mL concentration was added which was then allowed to gel in the 35 mm cell culture dishes for half an hour. Afterwards, MFH was performed for 1 h at 15.3 kA/m magnetic field with 330 kHz frequency in 2 × 2 turns Helmholtz coils.

## Results and discussion

### Magnetic fluid properties

**Figure 2:**
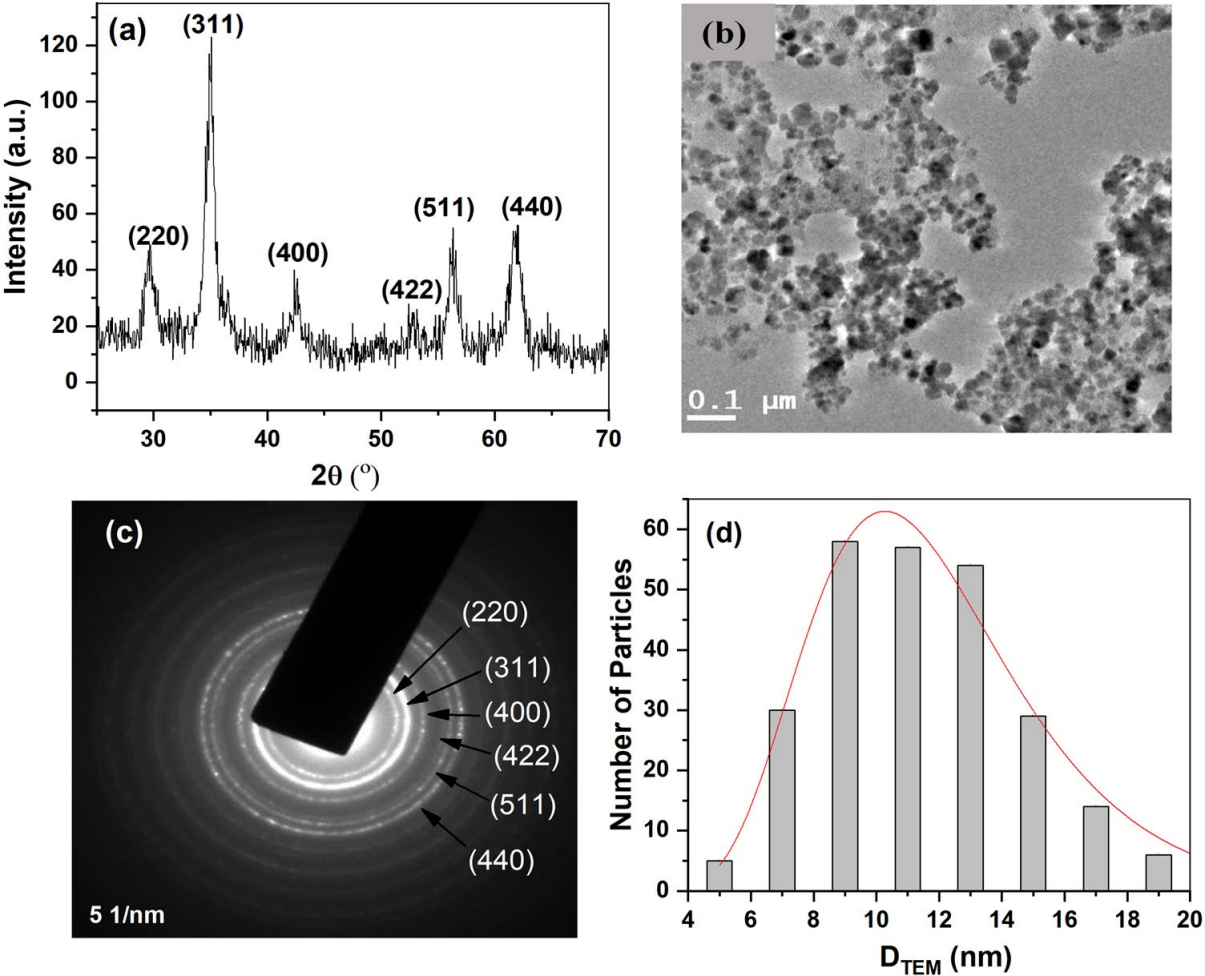
(**a**) XRD measurement, (**b**) TEM image of particles, (**c**) TEM diffraction pattern and (**d**) TEM size distribution.

Figure 2a shows the XRD pattern of synthesized MNPs. The crystal structure was found to be single phase spinel ferrite. Since there were no extra peaks other than ferrite phase, the particles were considered single phase, crystalline in nature and without any impurity. The crystallite size (D_crystallite_) was calculated using most intense peak (311) and Scherer formula^23^.

2 $${D}_{crystallite}=\frac{0.9\uplambda }{\mathrm{Bcos\theta }}$$

where B being full width half maxima of the most intense peak, θ is Bragg’s angle and λ is X-ray wavelength. The crystallite size was determined as 10.7 ± 0.5 nm. Lattice parameter ‘a’ was calculated using the analytical method^24^ and found as 0.8483 ± 0.0005 nm. X-ray diffraction peak positions were detected as (220), (311), (400), (422), (511) and (440) and the corresponding 2θ as 29.7°, 35.0°, 42.6°, 52.8°, 56.3°, and 61.8° respectively.

Figure 2b shows the TEM image of the particles. It is seen that particles are almost spherical in nature. Figure 2c shows the diffraction pattern studied using TEM. The electron diffraction pattern corresponding to the bright field image shows that the prepared MNPs possess a cubic spinel structure as confirmed by XRD. Nearly 300 particles from various TEM images were considered for plotting the histogram which is shown in Fig. 2d. The histogram was fitted with the log-normal distribution curve (red line) that resulted in a size of 11.3 ± 0.2 nm with size distribution of 0.31.

**Figure 3:**
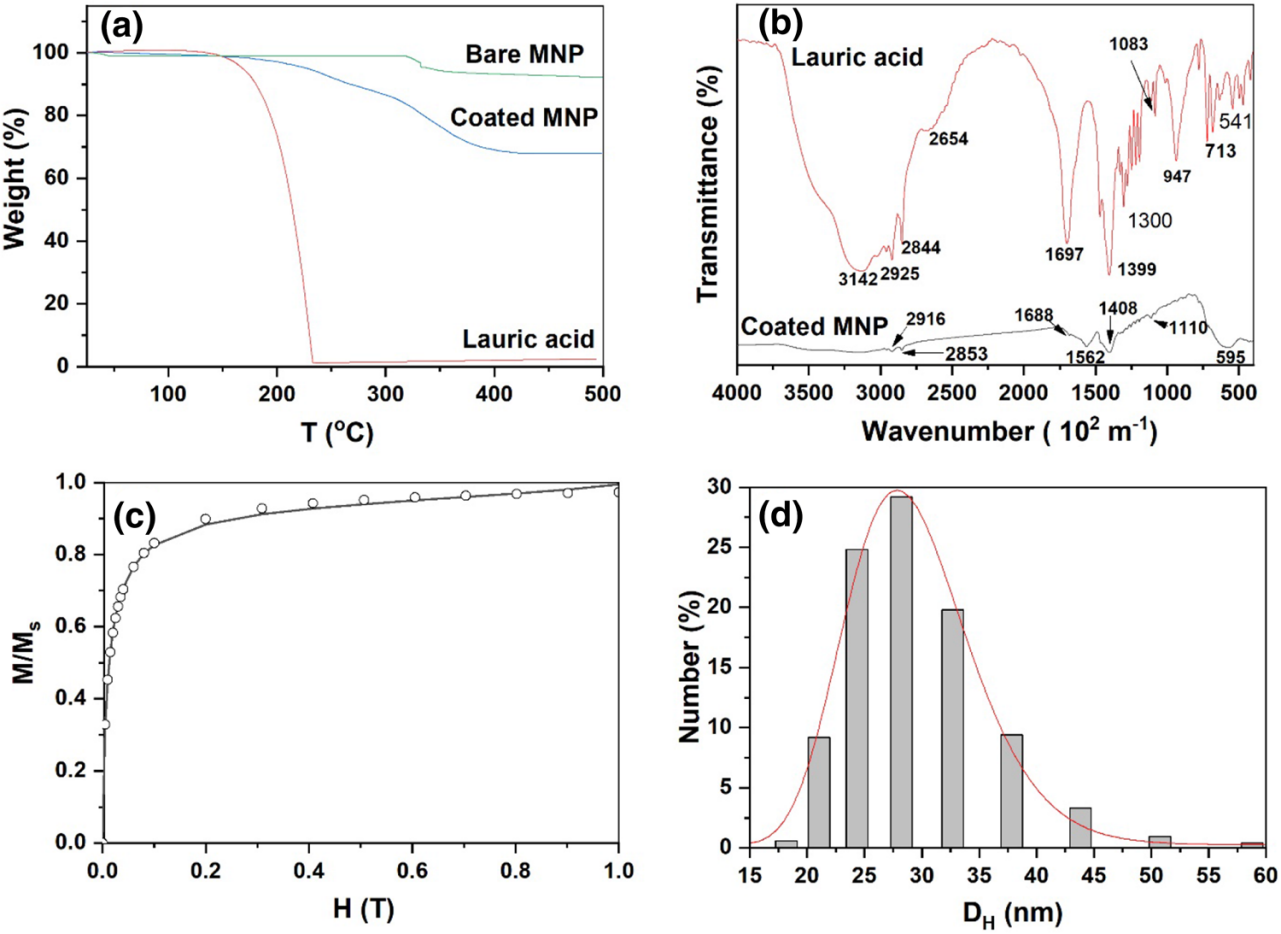
(**a**) TGA of bare particles, pure surfactant and surfactant coated particles, (**b**) FTIR measurement of coated particles and surfactant (Lauric acid), (**c**) Magnetization curve of magnetic fluid and (**d**) DLS size distribution of particles’ dispersion in Milli-Q water.

The bare as well as coated particles showed a total weight loss of 8% and 32.2%, respectively while for the surfactant, lauric acid, 98.6% weight loss was observed (Fig. 3a). For bare particles, the weight loss is due to removal of the water molecules or moisture adsorbed on the surface of particles which can be removed at relatively low temperature (< 373 °K). For lauric acid, weight loss (98.6%) occurs in a single step transition between 400 and 505 °K due to the decomposition of lauric acid molecule^25^. For coated MNPs, the transition temperature was shifted to higher temperature side and unlike pure surfactant, coated particles showed the major weight loss occurring in two steps; one at 516.9 °K and other at 604.4 °K. The high temperature transition indicates that the surfactant is chemically attached on the particle surface. The lauric acid molecules can be considered as arranged in bilayer fashion, namely, primary and secondary layers^26,27^. In the primary layer, the carboxylic acid groups of lauric acid are chemisorbed to the ferrite surface and the secondary lauric acid molecules are physically attached on the primary lauric acid molecules. The first weight loss of 10% may be attributed to the secondary/outer layer of lauric acid, while the second transition with 22.2% weight loss can be attributed to the decomposition of chemically bound lauric acid from the particle surface.

Figure 3b shows the FTIR spectra of pure surfactant and surfactant coated particles. The intense peak observed at 1698 * 10^2^ m^−1^ of the lauric acid curve is from the C=O stretch of the carboxylic head of the surfactant molecule. In the coated particles, this peak was shifted to 1702 * 10^2^ m^−1^ indicating that the lauric acid was strongly adsorbed or covalently bonded to the particle surface. The iron oxide Fe–O stretching vibration occurs at 595 * 10^2^ m^−1^ ^27^. For coated particles, absorption peaks at 2924 * 10^2^ m^−1^ and 2856 * 10^2^ m^−1^ corresponded to the asymmetric and the symmetric CH_2_ stretching, respectively which shifted from 2916 * 10^2^ m^−1^ and 2852 * 10^2^ m^−1^, respectively in the spectra of lauric acid. The results of the significant shift of these specific peaks to the lower frequency indicated that the hydrocarbon chains in the monolayer surrounding the nanoparticles can be in a closed-packed manner. The band at 930 * 10^2^ m^−1^ in the spectra of lauric acid was a characteristic band corresponding to O–H bending vibration. This band was completely vanished in the spectra of MNP. The weak band over 1689 * 10^2^ m^−1^ in the spectra of coated MNP could be due to the secondary layer of surfactant^28^. The difference of peaks at 1560 * 10^2^ m^−1^ and 1400 * 10^2^ m^−1^ indicates the existence of a bidentate structure or bidentate chelation that two oxygen atoms of the carboxylic group are attached to the surface of iron atom. From the above observation, we confirm that lauric acid was chemisorbed onto the particle surface.

Figure 3c shows the normalized magnetic response as a function of magnetic field for the parent fluid measured at room temperature. The magnetic parameters like domain magnetization, M_d_, magnetic particle size, D_m_, size distribution, σ and saturation magnetization, M_s_ were calculated from the Langevin fitting of the obtained data^29^ by assuming that the system was non-interacting and superparamagnetic. The best fitted value was obtained as D_m_ as 10 nm, M_s_ as 4 kA/m, M_d_ as 350 kA/m and σ as 0.62. The absence of remanence, coercivity and hysteresis, shows a typical characteristic of nanoparticles which are superparamagnetic at room temperature. The magnetization does not saturate at 1 T, suggesting the existence of a spin disorder at the surface of the nanoparticles. The surface of the nanoparticles is considered to be composed of some canted or disordered spins that prevent the core spins from aligning along the field direction resulting in decrease of the saturation magnetization of the small sized nanoparticles^30^.

The typical number distribution of magnetic fluid dispersed in Milli-Q water is shown in Fig. 3d. The number-weighted hydrodynamic distribution from DLS data obeys log normal distribution function (line in Fig. 3d). The results show polydispersed spherical particles with average diameter of 28.9 ± 0.1 nm. The width of the distribution curve (σ) was found to be 0.19. The higher value of hydrodynamic diameter as compared to the crystallite size obtained from XRD could be due to the multiple layers of coating of surfactant as well as possibility of formation of stable small aggregates upon dilution. Also, the equivalent sphere diameter measured by light scattering is generally higher than the actual hydrodynamic diameter of coated particle or the particle size measured from the other technique (XRD or TEM)^25^.

### Induction heating of magnetic fluid in cell culture medium

From the measured temperature rise with time, SAR value was calculated using the equation:

3 $$ SAR = C_{p} \cdot \frac{\Delta T}{{\Delta t}} \cdot \frac{1}{{\varphi_{magnetic} }} $$

where $\frac{\Delta T}{\Delta t}$ is the change in temperature with time, i.e., slope of the graph between temperature rise and time of induction heating, φ_magnetic_ is the weight fraction of magnetic content of nanoparticles and C_p_ is the specific heat capacity of the system (particles + carrier) given by,

$${C}_{p}={m}_{magnetic}*{C}_{p-particles}+{m}_{carrier}*{C}_{p-carrier}$$

where C_p_ for carrier and particlesis considered as 4.187 J/g K and 0.67 J/g K, respectively. The m_magnetic_ and m_carrier_ defines weight fraction of particles and carrier, respectively. The φ_magnetic_ is calculated as the ratio of mass of magnetic ions to the mass of total formula unit, which in the present case is 0.696.

**Figure 4:**
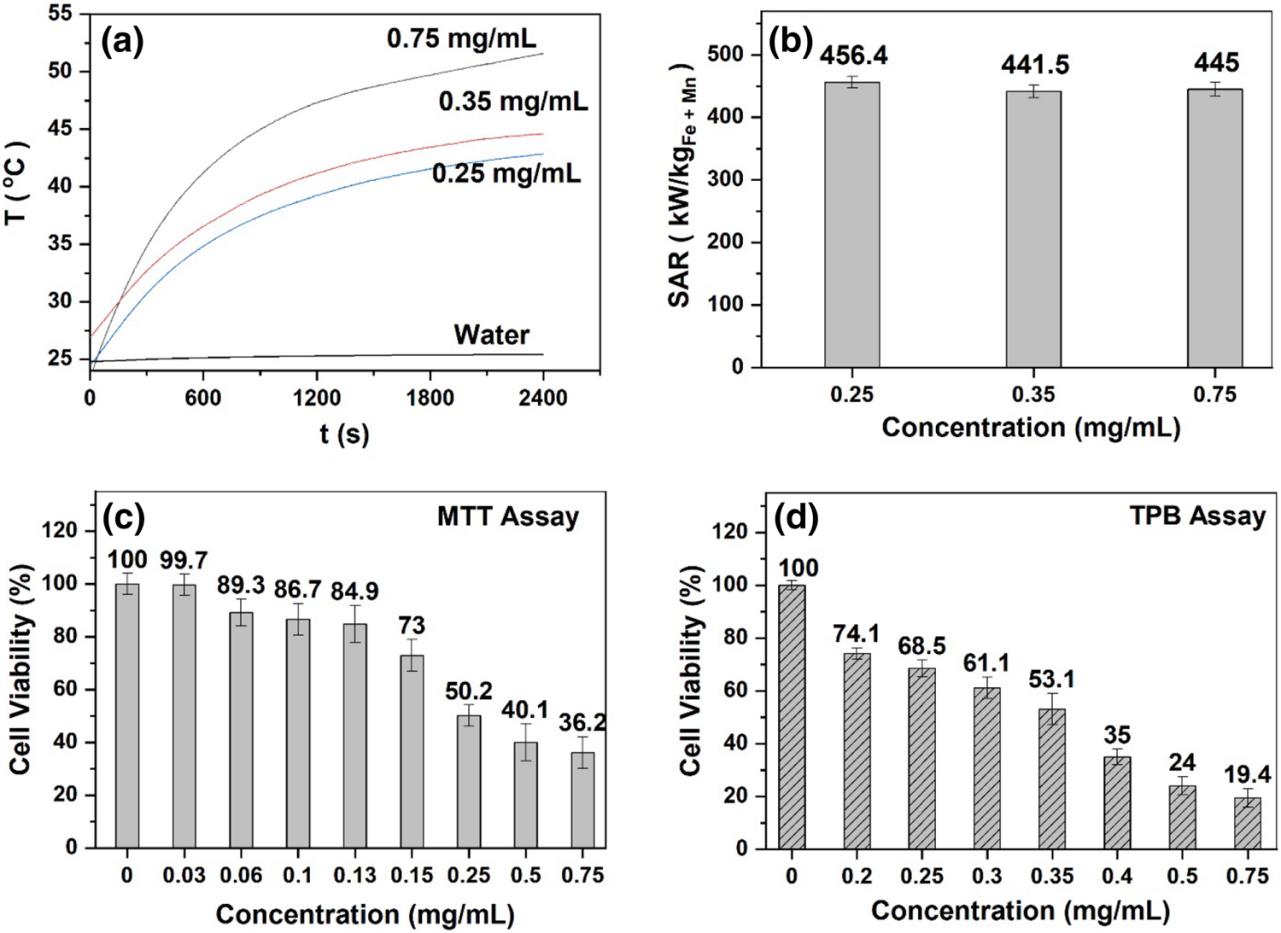
(**a**) Temperature versus time for different concentrations of MF diluted in cell culture media at 15.3 kA/m magnetic field, 330 kHz frequency and (**b**) corresponding specific absorption rate as a function of concentration. (**c**) MTT assay performed in 96 well plate and (**d**) Trypan blue assay performed in culture dishes on HeLa cells using varied magnetic fluid concentration to obtain the IC_50_ value_._

Figure 4a and b respectively shows the temperature rise versus time and corresponding SAR values for the magnetic fluid diluted in media (DMEM) at fixed frequency and magnetic field (330 kHz and 15.3 kA/m). Since the experimental set-up is not perfectly adiabatic, the slope of temperature versus time gets affected and under this condition the best way is to fit the data with Box–Lucas model for the whole curve^32^ described as T(t) = A (1 − exp(− Bt)). Here, T is temperature, t is time, A is saturation temperature and B is a parameter related to the curvature of the heating curve. The product A × B at t = 0 is the rate of change of heat and is equivalent to the ΔT/Δt ratio used for calculating the SAR. For a given weight fraction of 0.25 mg/mL and above mentioned value of specific heat capacity as well as φ_magnetic_, the SAR value was calculated using Eq. (3). The maximum SAR was found as 456.4 kW/kg_(Fe+Mn)_ for 0.25 mg/mL concentration. Almost three times higher value of SAR was detected for media-based fluid as compared to water-based fluid for the same concentration of particles, which could be attributed to the well dispersion of particles in media as compared to water upon dilution^33^.

### Effect of MF on cell viability

To study the effect of MF on cell viability and to identify the minimum inhibitory concentration of MF affecting 50% of cell population, we performed MTT^34^ and Trypan blue^35^ assays. Though MTT assay, based on cells’ metabolic response, is less laborious and quick to perform, TPB assay was simultaneously performed to visualize the cell death under a microscope. The cell viability was calculated as follows:

$$ \begin{aligned} Cellviability \,\left( \% \right) &= \hfill \\ &\frac{average\,absorbace\,from\,treated\,cells\,or\,average\,number\,of\,live\,cells\,after\,MF\,treatment\,in\,triplicates}{{average\,absorbance\,from\,control\,cells\,or\,average\,number\,of\,live\,+ dead\,cells\,in\,triplicates}} \times 100 \hfill \\ \end{aligned} $$

The formula is a combined presentation for both MTT and Trypan blue assay. The absorbance represent MTT assay while dead and live cells represents Trypan blue assay. The results of the effect of different concentration of MF on the viability of HeLa cells using MTT and TPB assays have been depicted in Fig. 4. Further, by employing the dose–response curve, the assays revealed IC_50_ of 0.27 and 0.3 mg/mL by MTT and TPB assays respectively. The IC_50_ of the present study on HeLa cells is in agreement with the results of Pradhan et al.^27^, who reported an approximate 0.4 mg/mL IC_50_ using nanoparticles of similar composition (MnFe_2_O_4_) on mammalian hamster kidney BHK21 cell line. Subsequently, to look into the effect of MFH, we selected three MF concentrations of 0.25, 0.35 and 0.75 mg/mL. However, the lesser concentration of 0.25 mg/mL was not sufficient enough to obtain the hyperthermia temperature (43–45 °C), as evident from the induction heating results shown in Fig. 4a. Therefore, we proceeded with a little higher concentration than IC_50_ value, i.e., 0.35 mg/mL. The reason to choose this concentration was to reach as well as maintain the hyperthermia temperature between 43 and 45 °C (Fig. 4a) to study MFH. The induction heating was also conducted on water to determine if heating is being caused by MNPs and not because of the heating of the coil due to excessive flow of current through the coil. The same is reflected in Fig. 4a where it is seen that the temperatures rise in water is less than 1 °C even after prolonged heating of the coil.

The MFH was performed under induction heating at 15.3 kA/m field, 330 kHz frequency for 30 and 60 min. In our earlier work^33^, we observed the cytotoxicity on HeLa cells at 0.75 mg/mL MF concentration using MTT assay with 30 and 60 min hyperthermia. The results showed significant decrease in cell viability, up to 36.2% due to MF alone that was further enhanced to 30.4% and 24.5% after 30 and 60 min of HT sessions, respectively. A high cell death detected due to 0.75 mg/mL MF concentration without induction heating can be attributed to higher concentration of fluid, formation of particle aggregates^31^ in the media leading to change in the actual concentration of MNPs, and/ or toxicity of the surfactant. These factors would have led to a high toxic environment resulting in cell death. To confirm the possible formation of aggregates in the fluid, we performed DLS experiments by diluting the MF in water as well as in cell culture media at the concentration of 0.75 mg/mL. The hydrodynamic size was found as 28.9 nm and 64 nm respectively for the water and media (data not shown). DLS result indicated the possibility of formation of small aggregates in presence of ions and proteins in the media which is in line with the previous report of Chanteau et al.^36^. Therefore, we analysed the hyperthermic effects of MF at 0.35 mg/mL concentration.

**Figure 5:**
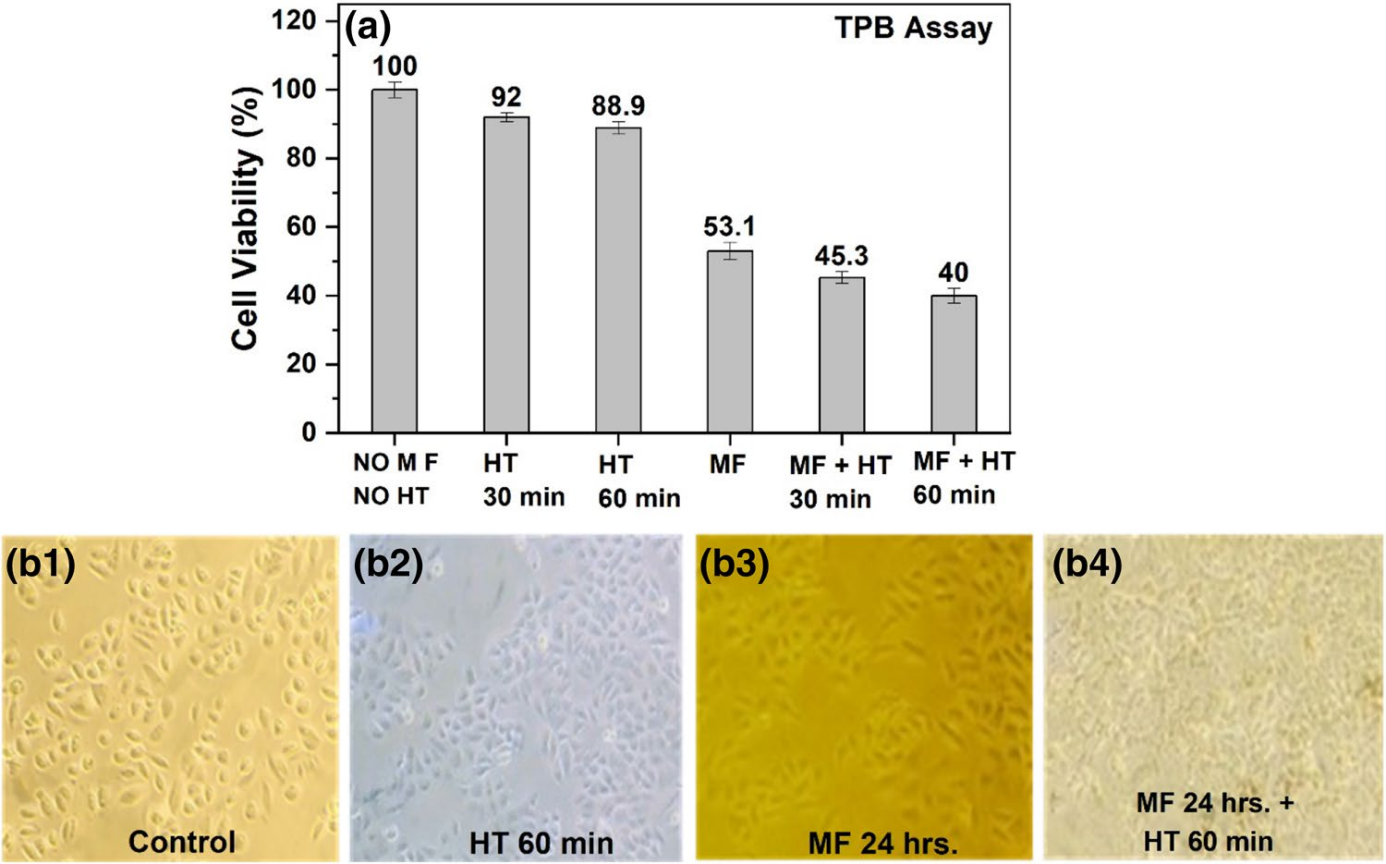
(**a**) HeLa cells’ viability results using Trypan blue assay with and without 0.35 mg/mL concentration magnetic fluid for 24 h interaction and magnetic induction heating for 30 min and 60 min. All the groups showed significant cell death compared to untreated cells (no MF and HT) (*p* < 0.01). Microscopic image of HeLa cells (**b1**) control, (**b2**) after 60 min hyperthermia (**b3**) with magnetic fluid interaction after 24 h and (**b4**) with MF of 24 h interaction and hyperthermia treatment of 60 min.

The cytotoxicity at 0.35 mg/mL concentration of magnetic fluid revealed an approximate 50% reduction in viability of HeLa cells after 24 h of MF treatment alone, that was further reduced to 45 and 40% after 30 and 60 min of HT sessions respectively (Fig. 5a). Figure 5(b1) shows the microscopic image of untreated HeLa cells without MF and HT and Fig. 5(b2) after 1 h of HT treatment without MF, revealing very mild effect of HT solely on cell proliferation. This indicates that the cell tolerated heat shock effect for survival. However, a reduced cell count and change in cell morphology was observed after 24 h MF treatment (Fig. 5-b3) that was slightly increased when the cells were also treated with 1-h HT (Fig. 5-b4) that revealed the beginning of the effect of MF combined with HT on cell survival and showing a significant difference in cell viability compared to control cells (*p* < 0.01).

**Figure 6:**
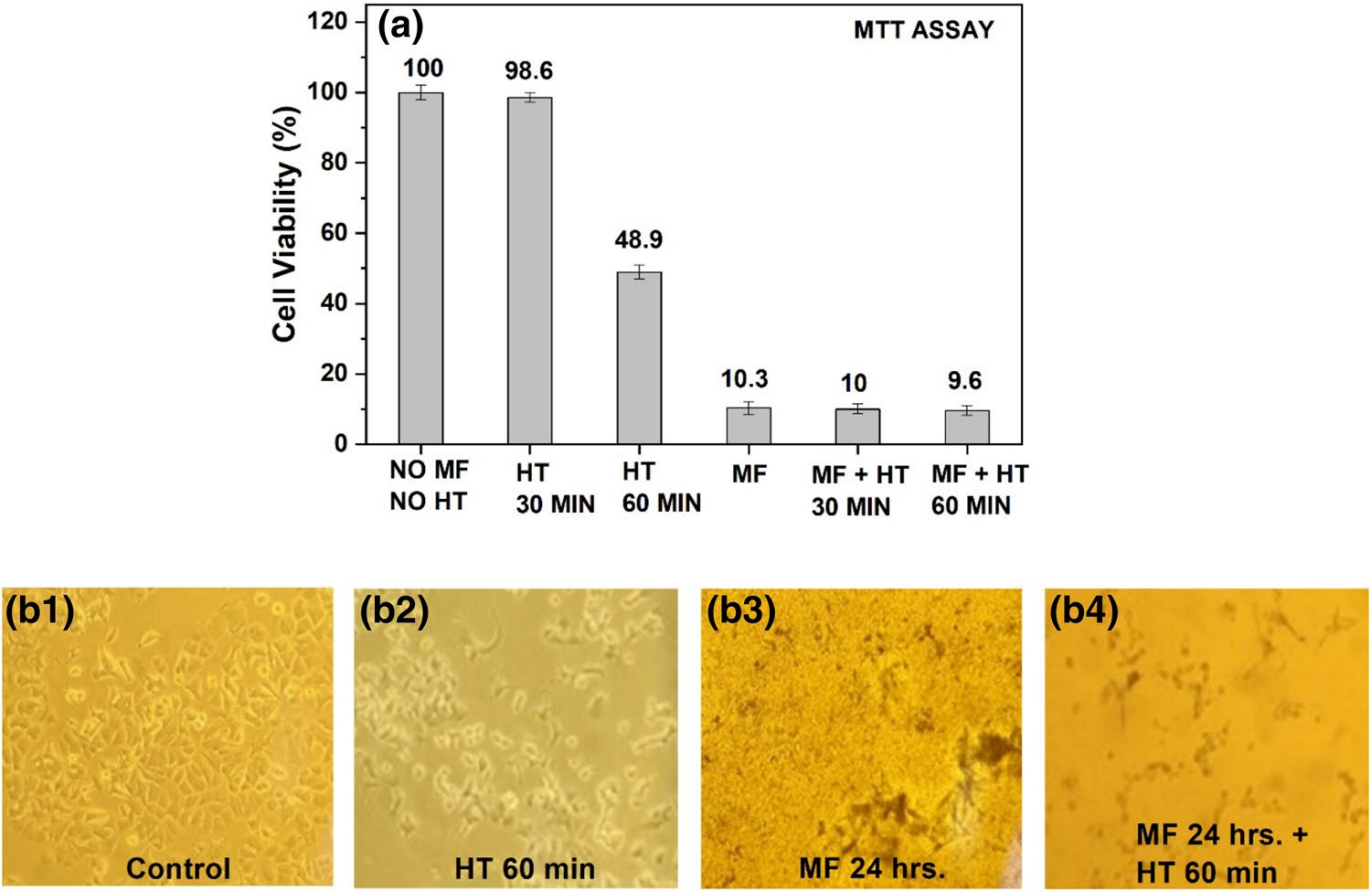
(**a**) MCF7 cells’ viability results using MTT assay with and without 0.35 mg/mL concentration magnetic fluid for 24 h interaction and magnetic induction heating for 30 min and 60 min. All the groups showed significant cell death compared to untreated cells (no MF and HT) (*p* < 0.01) except HT 30 min group. Microscopic image of MCF7 cells (**b1**) control, (**b2**) after 60 min hyperthermia, (**b3**) negligible live cells with magnetic fluid interaction after 24 h and (**b4**) negligible live cells with 24 h MF interaction and hyperthermia treatment of 60 min.

Furthermore, our preliminary hyperthermia examination on MCF7 cells at 0.35 mg/mL concentration of magnetic fluid revealed an increased sensitivity of breast cancer cells against hyperthermia during initial phase itself where a 60 min HT without MF led to more than 50% cell death compared to control cells (*p* < 0.01). The viability of MCF7 cells was drastically reduced to approximately 10% after treatment with MF as well as MF with 30 and 60 min HT (*p* < 0.01) (Fig. 6a). The MCF7 cells also showed significant cell death (*p* < 0.01) compared to HeLa cells after 60 min HT treatment without MF as well as with MF treatment of different time intervals. Figure 6(b1) shows the microscopic image of untreated MCF7 cells and Fig. 6(b2) after 1 h of HT treatment without MF, revealing visible cell death due to HT only. Further, an extreme reduction in cell viability and severe morphologic changes can be observed after 24 h of MF treatment alone (Fig. 6-b3) as well as after MF with HT treatment (Fig. 6-b4). These results indicate that breast cancer cells are possibly more prone to HT as well as MFH as compared to cervical cancer cells. This may be attributed to the physiologic difference of two cell types. Moreover, cytotoxicity due to MNPs’ surfactant cannot be denied. Detailed investigations on different hormone-dependent and non-dependent cancer cell lines originating from diverse body tissues are warranted.

Similar studies have been performed by various researchers using different types of ferrite based MNPs for hyperthermia applications. Villanueva et al.^37^ used silica coated La-Sr-Mn ferrite on HeLa cells. They reported that three-hour incubation with MNPs at 0.5 mg/mL concentration produced 20% cell death after hyperthermia for 30 min. While hyperthermia without MNPs and MNPs alone did not cause cell death greater than 10% as the temperature rise was only 0.5 °C (from 37 to 37.5 °C). The hyperthermic cell death is comparable to results from our studies. Gkanas^38^ performed magnetic fluid hyperthermia on HeLa cells using polyethylene glycol and triethylene glycol coated iron oxide MNPs at 2 mg/mL concentration, whereas the concentration of our magnetic fluid was comparatively less (0.35 mg/mL). Their result of cytotoxicity on control cells treated with MNPs but without hyperthermia is in line with our observation of approximate 50% cell death under similar conditions. Further, to achieve more than 90% cell death, they treated cells for 48 h with MF before hyperthermia and to maintain the temperature window of 42–46 °C, manually switched off the applicator after 2–3 min. In our case, the hyperthermia temperature window of 43–45 °C was maintained for 60 min without altering the applicator parameters; which was achieved by optimizing the concentration of our novel temperature sensitive nature of the particle composition. Our observed cell death due to magnetic hyperthermia was approximately 60%.

Makridis et al.^18^ used manganese ferrite MNPs on human osteosarcoma (Saos-2) cells at 0.25 and 0.5 mg/mL concentration with hyperthermic duration of 6 min and 41–45 °C reaching in 4–6 min. This resulted in 25–30% cell death due to magnetic fluid hyperthermia and only 5% cell death due to hyperthermia alone. These results are matching with our observations on HeLa cells. Further, hyperthermia for longer durations was performed by Bohara et al.^39^ using Co-Zn ferrites at 1 mg/mL concentration on MCF7 cells that led to approximate 62% and 67% cell death after 30- and 60- min of magnetic hyperthermia respectively, while maintaining temperature window of 42–45 °C. Patil et al.^40^ used Fe_3_O_4_ particles coated with oleic acid and betaine HCl at 1 mg/mL concentration on mouse fibroblast L929 and HeLa cells. Their results showed approximately 70, 80 and 95% cell death at 30, 60 and 90 min hyperthermia respectively where temperature was maintained at 44–45 °C. Ours as well as previous results suggest that the concentration of magnetic fluid and duration of hyperthermia are important parameters towards effective hyperthermic death of cells that warrants further investigation.

As we observed that MFH at 0.35 mg/mL concentration was not sufficient to cause absolute reduction in the viability of the cell population, we performed multiple sessions hyperthermia, where the cells under the same concentration were grown up to 72 h and received single, double and treble times 1-h hyperthermia treatment. After every hyperthermia session, the cells were incubated for 24 h to relax and regain their growth properties before a subsequent hyperthermia session.

**Figure 7:**
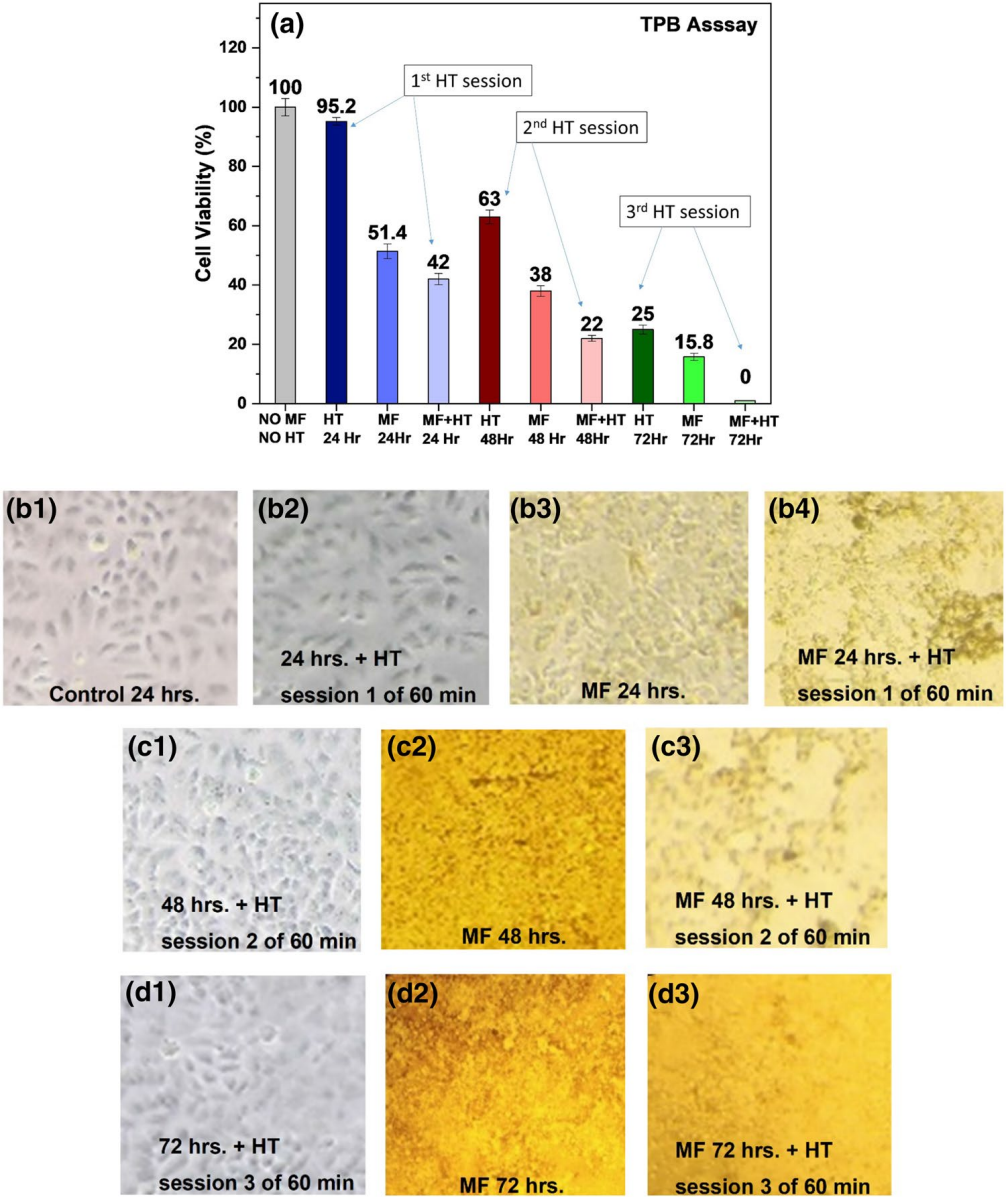
Multiple sessions hyperthermia at 0.35 mg/mL MF concentration. (**a**) HeLa cells’ viability using Trypan blue assay after 24, 48 and 72 h treatment. Cell death was statistically significant in the different groups compared to control group (*p* < 0.05; *p* < 0.01). Morphology of HeLa cells (**b1** to **b4**) after 24 h, (**c1** to **c3**) after 48 h and (**d1** to **d3**) after 72 h.

In the first 24 h, the cells of the first set (as described in the methods section) that underwent one-hour HT only, showed 95% cell viability followed by ~ 50% viability by cells that were exposed to MF only but without HT, compared to controls. However, the cells, when treated with both MF and 1-h HT session showed almost 60% reduction in cell viability. Further, after 48 h, the cells of second set showed reduced cell viability up to 63% after second hyperthermia treatment alone. Presence of MF alone led to 38% cell viability that was further reduced to 22% due to the combined effects of MF as well as two hyperthermia sessions. Greater enhancement of cell death took place after 72 h, where the cell viability was reduced to 25%, 15.8% and 0% due to three hyperthermia sessions without MF, MF alone, and MF along with three HT sessions respectively. Finally, the multiple sessions HT revealed a certain death of cell population after 72 h treatment of MF along with three HT sessions of 1 h each after every 24 h (Fig. 7). In all the cases, cell death was statistically significant when compared to control cells (*p* < 0.05; *p* < 0.01). Multiple sessions hyperthermia was also performed by Makridis et al.^18^ on Saos-2 osteoblast cell line using Mn ferrite particles with a difference of 48 h between two sessions. They reported up to 90% cell death at second hyperthermia session after 72 h of MF treatment. Our results indicate that hyperthermia due to MF with induction heating in short multiple sessions over a long period of time is remarkably effective in completely killing the cancer cells in vitro. However, cell death through apoptosis leading to efficient killing of cancer cells is desirable. Therefore, based on the present study results the further investigations are warranted on refining the dose and time leading to cancer cell death by apoptosis.

### Hyperthermia in agarose phantom system

**Figure 8:**
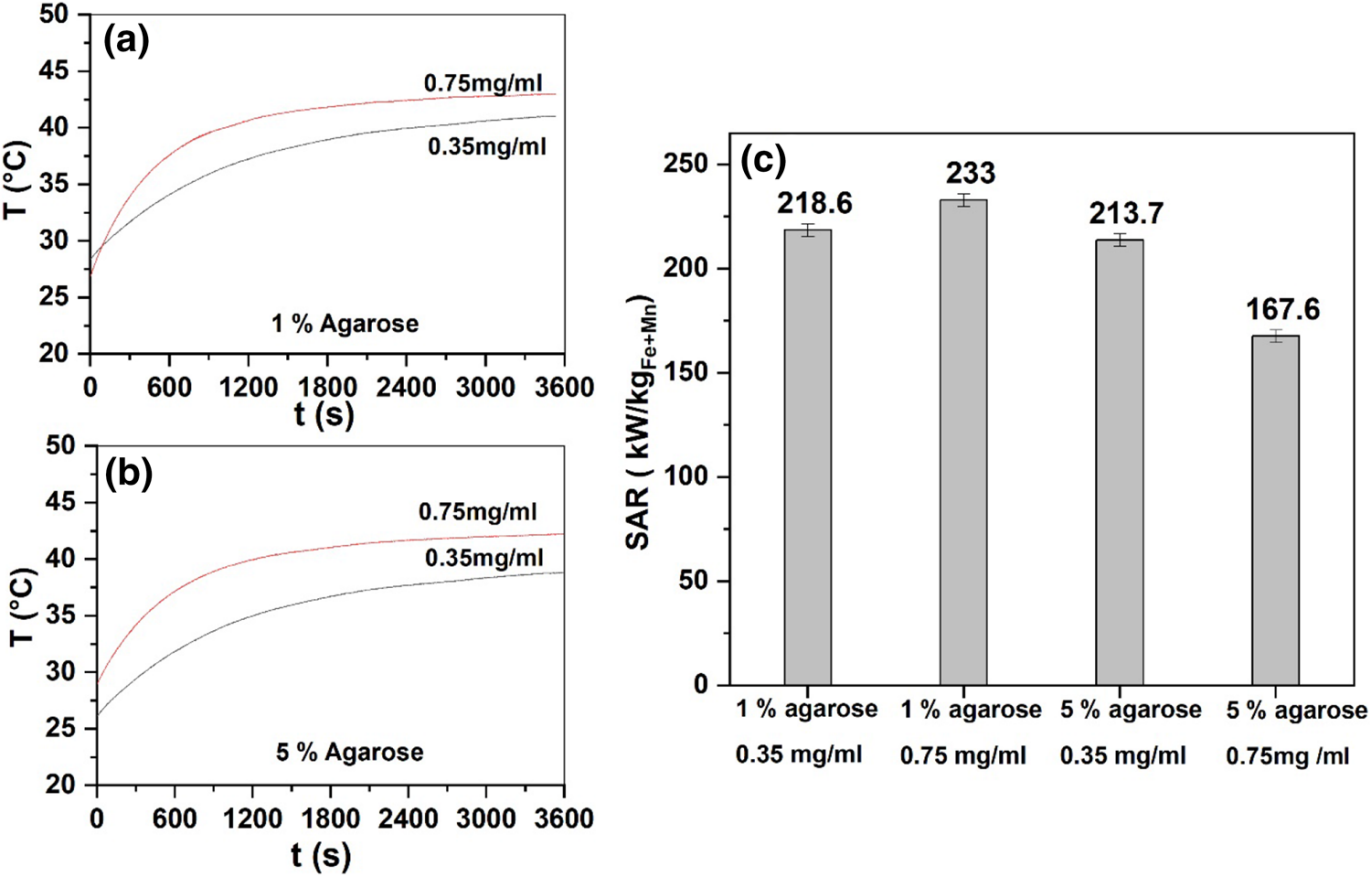
Temperature versus time at 0.35 and 0.75 mg/mL MF concentration in (**a**) 1% and (**b**) 5% agarose, (**c**) corresponding specific absorption of diluted magnetic fluid.

When injected into a tumor, MNPs may be immobilized and the Brownian rotation may be suppressed during MFH treatment. Since agarose gel mimics cell’s viscous environment, MFH was performed to reproduce in vivo conditions of tumor tissues with different porosity and viscosity. One and 5% agarose gels were used and the rise of temperature versus time and corresponding SAR values were plotted for 0.35 and 0.75 mg/mL magnetic fluid concentration as shown in Fig. 8. The rise of temperature in the agarose system was low as compared to media plus MF system which provides a rough approximation/simulation of in vivo experiment. We observed an almost similar SAR for 1% agarose gel environment at 0.35 mg/mL concentration of magnetic fluid whereas slight decrease in SAR was observed for 5% agarose gel at 0.75 mg/mL concentration. However, a drastic decrease of 50–60% was detected when compared to media plus MF system (please refer Fig. 4b). A similar 50% decrease in SAR was also reported by Makridis et al.^18^ and Shah et al.^41^ for ferrite-based near 10 nm size MNPs and 1.5% agarose gel system. The decrease in specific absorption rate for agarose gel can be attributed to hindered Brownian movement of particles due to agarose gel. Also, MNPs in water-based solutions can form chain-like structures causing higher SAR but not when immobilized in a gel^10^. A similar experiment was performed by Avolio et al.^42^ where they used 0.5% and 2% agarose gel and reported decrease in SAR for 14 and 18 nm ferrite MNPs, however they found an almost similar SAR for 10 nm particles when compared to aqueous MNP system. Similarly, Chen et al.^43^ observed 47% reduction in SAR of MNPs from the original value for rigid silicon-based organic polymer. Together, these observations suggest that actual MNPs concentration required for in vivo experiments may be higher as compared to in vitro experiments.

## Conclusions

We report here for the first time, synthesis of Mn–Zn ferrite nanoparticles based self-regulating temperature-controlled magnetic fluid as well as its effect on cervical and breast cancer cell death using hyperthermia. The particles were spherical in shape with 10.7 ± 0.5 nm crystallite size. The size obtained from TEM was 11.3 ± 0.2 nm with size distribution of 0.31. The FTIR and TGA confirmed that the surfactant was chemically attached on the particles’ surface. The temperature window of 43–45 °C was maintained for 1 h at smallest possible concentration of 0.35 mg/mL magnetic fluid concentration without altering the magnetic field applicator parameters. Single session MF hyperthermia did not significantly kill cancer cells. Moreover, as cancer cells tend to resist treatment and show re-growth, to obtain complete cell death, the multiple sessions hyperthermia was investigated using a 24 h window till 72 h using TPB assay, which resulted in complete death of cell population. Further, to simulate an in vivo cellular environment, a phantom consisting of MNPs dispersed in 1 and 5% agarose gel was also constituted and studied. Results showed an almost 50–60% decrease in SAR of agarose system as compared to media plus MF system that may be attributed to hindered Brownian movement of particles due to agarose gel. We observed excessive cytotoxicity of magnetic fluid even without induction heating that may be due to the effect of surfactant layer used in preparing magnetic fluid. Magnetic fluid preparation with less toxic surfactants is desirable. Our study also suffered from the limitation of analyzing MFH effect on the cancer cells of varied pathophysiology originating from different tissue types. Another drawback was the non-comparison of multiple sessions hyperthermia between HeLa and MCF7 cells. Nevertheless, our results suggest that the concentration of magnetic fluid as well as duration of hyperthermia are important parameters towards effective killing of cancer cells and higher MNPs concentration may be required at in vivo level. The study outcomes may aid in deciding the future cancer therapeutic strategies using temperature sensitive magnetic fluid-based hyperthermia.
